# Clinicians’ and womens’ experiences of two consent pathways in a trial of timing of clamping at very preterm birth: a qualitative study

**DOI:** 10.1186/1745-6215-16-S2-P70

**Published:** 2015-11-16

**Authors:** Susan Ayers, Alex Sawyer, Celine Chhoa, Angela Pushpa-Rajah, Lelia Duley

**Affiliations:** 1 Centre for Maternal and Child Health Research, City University, London, UK; 2 Nottingham Clinical Trials Unit, University of Nottingham, Nottingham, UK

## Background

Recruitment to trials when birth is imminent requires offering consent at a difficult and stressful time, often with limited time. The Cord Pilot Trial assessed timing of cord clamping at very preterm birth. To ensure high risk women were not excluded we developed a two stage oral assent pathway, for use when birth was imminent. A third of women were recruited using this pathway. The aim of this study was to explore clinicians’ and women’s’ experiences of the two consent pathways.

## Methods

A qualitative interview design with semi-structured interviews. Clinicians and women were recruited from the 8 trial sites. Results were analysed using systematic thematic analysis.

## Results

17 clinicians were interviewed, 11 had experience of both pathways and 6 of one stage written consent only. [DL1]Themes identified: consent as a continual process; consent as a record versus consent as a legal document; team approach; different consent pathways for different trials; balance between time and information.

23 women were interviewed, 5 had been offered oral assent and 18 one stage written consent. Themes identified: importance of staff; time and information; reasons for agreeing to consent; trial secondary in women’s minds; understanding randomisation.

## Conclusion

Overall, clinicians thought that one stage written consent was optimal for offering consent but were positive about the use of oral assent when there is limited time for offering participation. Women were positive about their experiences, particularly about the staff who approached them. Nevertheless, there were gaps in understanding of the trial in some women’s accounts.

